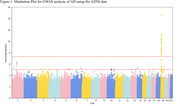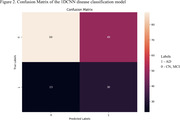# Deep‐learning‐based Polygenic Dementia Risk for Alzheimer’s Disease

**DOI:** 10.1002/alz.089059

**Published:** 2025-01-09

**Authors:** Linke Li, Metin Nafi Gurcan, Sheng Luo, Da Ma

**Affiliations:** ^1^ Duke University School of Medicine, Durham, NC USA; ^2^ Wake Forest University School of Medicine, Winston‐Salem, NC USA

## Abstract

**Background:**

Alzheimer’s Disease (AD) is a common form of dementia among the elderly with a large percentage of estimated heritability. Conventional polygenic risk analysis only accounts for the linearly combined genomic effects of single nucleotide polymorphisms (SNPs). On the other hand, a deep‐learning‐based approach is able to uncover nonlinearly associated effects from data with high‐dimensional features such as genomics. This study aims to derive deep‐learning‐based polygenic risk analysis for AD through deep‐learning‐based classification analysis.

**Methods:**

793 patients’ genetic data were extracted from the Alzheimer’s Disease Neuroimaging Initiative (ADNI) GO/2 cohorts genetic database, with sex, age, and dementia diagnosis data extracted from ADNI clinical data. After quality control, 760 patients remained in the study and their SNP data was then imputed. Genome‐wide association study (GWAS) was conducted to determine the chromosome for study. One‐dimensional convolutional neural networks (1D‐CNN) were trained to discern phenotype‐associated SNPs for developing disease classification models. Subjects were classified into either cognitively normal & mild cognitive impaired (N = 542), or diagnosed with AD (N = 212) during the study period. The categorical information of SNPs (0, 1, or 2 alleles) on chromosome 19, containing APOE genes, was used as the input features for 1D‐CNN‐based disease case classifier model, with a 75/25 train/test split to evaluate. The weighted cross‐entropy function was used for the imbalanced AD and non‐AD cases in the samples.

**Results:**

GWAS results showed strongest AD association for chromosome 19, which was selected for CNN dementia classification model training, while potential associated SNPs were also revealed on chromosome 1 (Figure 1). The model achieved area‐under‐the‐curve (AUC‐ROC) of 0.60. The sensitivity and specificity are 39.06% and 77.78%, respectively, and the balanced accuracy is 58.42%. Figure 2 shows the corresponding confusion matrix.

**Conclusion:**

The GWAS results reinforce the APOE region’s established association with AD. The 1D‐CNN’s model tended to misclassify diseased individuals, potentially due to the fact of including MCI patients with a potential risk of AD in the control group. Future work will include refining the model hyperparameter as well as collecting a more disease‐balanced dataset.